# Climate change, adaptation, and phenotypic plasticity: the problem and the evidence

**DOI:** 10.1111/eva.12137

**Published:** 2014-01-08

**Authors:** Juha Merilä, Andrew P Hendry

**Affiliations:** 1 Ecological Genetics Research Unit, Department of Biosciences, University of HelsinkiHelsinki, Finland; 2 Redpath Museum & Department of Biology, McGill UniversityMontreal, QC, Canada

**Keywords:** environmental change, evolution, genetics, global change, individual plasticity, natural selection

## Abstract

Many studies have recorded phenotypic changes in natural populations and attributed them to climate change. However, controversy and uncertainty has arisen around three levels of inference in such studies. First, it has proven difficult to conclusively distinguish whether phenotypic changes are genetically based or the result of phenotypic plasticity. Second, whether or not the change is adaptive is usually assumed rather than tested. Third, inferences that climate change is the specific causal agent have rarely involved the testing – and exclusion – of other potential drivers. We here review the various ways in which the above inferences have been attempted, and evaluate the strength of support that each approach can provide. This methodological assessment sets the stage for 11 accompanying review articles that attempt comprehensive syntheses of what is currently known – and not known – about responses to climate change in a variety of taxa and in theory. Summarizing and relying on the results of these reviews, we arrive at the conclusion that evidence for genetic adaptation to climate change has been found in some systems, but is still relatively scarce. Most importantly, it is clear that more studies are needed – and these must employ better inferential methods – before general conclusions can be drawn. Overall, we hope that the present paper and special issue provide inspiration for future research and guidelines on best practices for its execution.

## Introduction

Adaptive evolution occurs when the genetic constitution of a population changes as a consequence of natural selection. Thus, to demonstrate that adaptation has occurred, evidence is needed for genetic change and that natural selection has been the causal force. Given that the genetic underpinnings of most traits are still not known (Ellegren and Sheldon 2008; Mackay et al. 2009; Anderson et al. 2013) and that the accurate measurement of natural selection is difficult (Kingsolver et al. 2001, 2012; Kruuk et al. 2003; Hersch and Phillips 2004; Morrissey and Hadfield 2012), obtaining hard evidence to conclusively demonstrate adaptive evolution in the wild represents a major and ongoing challenge. Given these logistical difficulties, and following from the traditional view that natural selection is a powerful force (Endler 1986), a frequent default has been to infer adaptive evolution based simply on evidence that mean phenotypes have changed in ways that intuition suggests are adaptive. This is not enough.

One major reason the above intuition-based inferences are not sufficient is that phenotypic change can be the result of either genetic change or phenotypic plasticity, the latter occurring when individuals of a given genotype adjust their phenotype according to the conditions they experience (West-Eberhard 2003). Indeed, a number of phenotypic differences originally thought to be genetically based were subsequently attributed to phenotypic plasticity (e.g., James 1983; Charmantier et al. 2008; Teplitsky et al. 2008). In the wake of such demonstrations phenotypic plasticity is increasingly adopted as a parsimonious model that is to be rejected only if direct evidence is obtained for genetic change: That is, plasticity is treated as a null model. The reality, however, is that inferences regarding phenotypic plasticity also should be supported by a specific set of evidentiary criteria (see below).

Another major reason why the above intuition can fail is that phenotypic changes may or may not be adaptive. On the one hand, maladaptive or nonadaptive genetic changes can occur owing to genetic drift or gene flow, rather than natural selection. On the other hand, maladaptive or nonadaptive plastic changes might occur as a result of stress, nutrient limitation, and a host of other reasons (Gotthard and Nylin 1995; Grether 2005). In reality, then, genetic versus plastic changes and adaptive versus nonadaptive changes should be treated as alternative models to be compared based on the weight of evidence. In the present paper, we consider these issues in the context of contemporary global (climate) change.

The possibility of evolution in response to climate change started to gain attention in the late 1980s and early 1990s (e.g., Holt 1990; Lynch and Lande 1993; Bürger and Lynch 1995). Interest has since accelerated rapidly – in the last two decades more than 30 review and perspective articles have emerged on the topic (Merilä 2012), and 12 new reviews are published in this issue. At the same time, concerns have been voiced about the quality of inference: In particular, few studies have convincingly demonstrated that observed phenotypic shifts have a genetic basis (Gienapp et al. 2008; Merilä 2012). Likewise, despite the promise and enthusiasm associated with using molecular genetic and genomic approaches to detect climate-driven evolution (Hoffmann and Daborn 2007; Reusch and Wood 2008), few studies have been able to realize it (but see: Umina et al. 2005; Balanyá et al. 2006) and fewer still have been able to link genetic changes to phenotypes and to confirm that the changes are adaptive.

**Figure 1:**
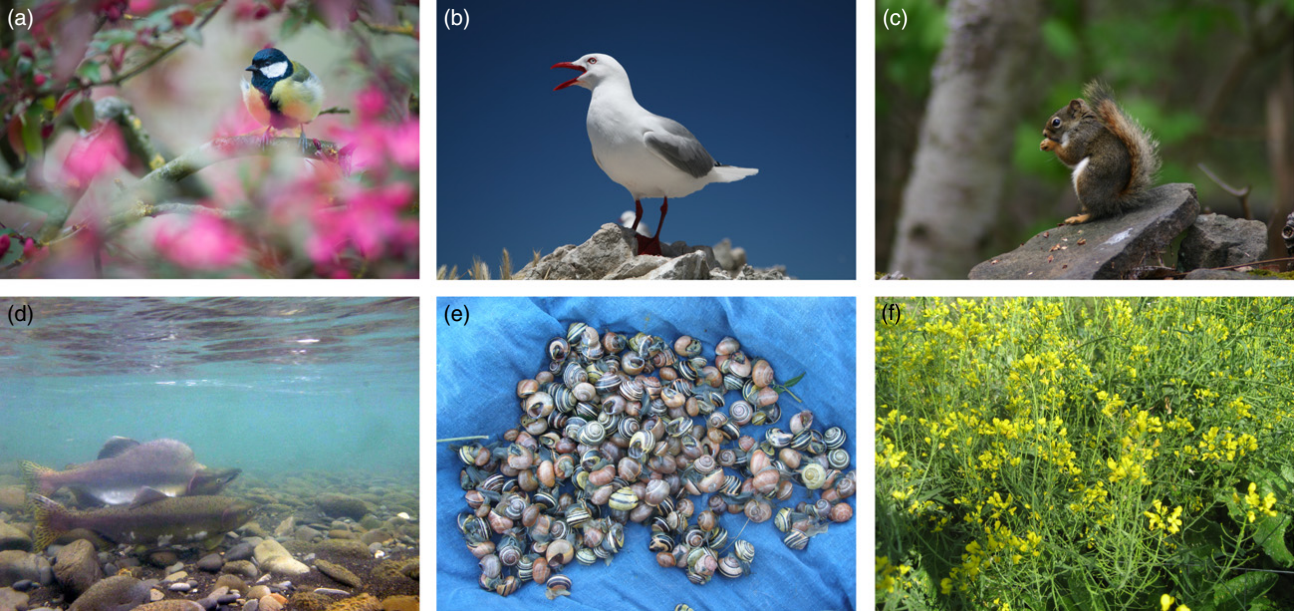
Examples of model species utilized in research on genetic underpinnings of climate change responses. (A) The great tit (*Parus major*), (B) red-billed gull (*Larus novaehollandiae*), (C) red squirrel (*Tamiasciurus hudsonicus*), (D) pink salmon (*Oncorhynchus gorbuscha*), (E) grove snail (*Cepaea nemoralis*) and (F) field mustard (*Brassica rapa*). Photograph credit: (A): S. Caro, (B): J. Merilä, (C): C. Kolacz, (D): A. P. Hendry, (E): M. Ozgo, (F): S. Franks.

In addition to the scarcity of evidence for genetic change, increasing support has emerged for its main alternative. That is, many studies have now reported a role for plasticity in shaping phenotypic responses to contemporary disturbances such as climate change (e.g., Charmantier et al. 2008; Gienapp et al. 2008; Hendry et al. 2008; Teplitsky et al. 2008). Partly for this reason, it has become increasingly common for authors to assume a role for plasticity unless direct evidence for genetic change has been obtained: that is, the null model approach mentioned above. In most cases, however, the lack of genetic evidence stems from the lack of actual tests for genetic change rather than concrete evidence that no genetic change has occurred. In such instances, all that can be stated with certainty is that the relative contributions of genetic change and plasticity are unknown. In addition, the general point made above that plastic responses might or might not be adaptive also applies to climate change. For instance, Teplitsky et al. (2008) provided evidence that climate-driven plastic decreases in the body size of red-billed gulls (*Larus novaehollandiae;* Fig. 1) were likely the result of environmental stress, rather than adaptive responses.

Beyond this ambiguity about genetic versus plastic changes and adaptive versus nonadaptive changes, another inferential difficulty comes in determining the specific environmental factor causing a particular phenotypic/genetic change (Endler 1986; Wade and Kalisz 1990; MacColl 2011). A well-known example of this problem is the difficulty in confidently ascribing phenotypic trends in fish stocks to the effects of fishing versus other environmental changes (e.g., Kuparinen and Merilä 2008). In the context of climate change, increasing temperatures are often coincident with many other environmental changes that shape phenotypic responses, whether genetic or plastic. Thus, the demonstration that a phenotypic change is genetic or plastic and adaptive or not does not necessarily provide confirmation that climate change, or more specifically climate warming, is the specific cause.

In the present paper, we outline approaches that have been used for making the above inferences in relation to climate change. Our major goal in doing so is to consider in detail what the various methods can and cannot tell us about proximate and ultimate causes. One of our key points is that evolution and plasticity (whether adaptive or not) should be treated as alternative models to be weighed against each other in light of data. That is, one alternative should not be considered a ‘null’ model to be assumed unless it can be rejected in favor of another.

After drafting the present paper, we provided it to experts who were well positioned to evaluate the strength of evidence for adaptive evolutionary responses to climate change in particular taxa (*viz*. fish, birds, mammals, amphibians and reptiles, marine animals and plants, terrestrial invertebrates, freshwater invertebrates, terrestrial plants, and marine phytoplankton) or in the context of particular topics (Bergmann's rule, theoretical models). The results of these assessments form the rest of this special issue (Boutin and Lane 2014; Charmantier and Gienapp 2014; Collins et al. 2014; Crozier and Hutchings 2014; Franks et al. 2014; Kopp and Matuszewskib 2014; Reusch 2014; Schilthuizen and Kellermann 2014; Stoks et al. 2014; Teplitsky and Millien 2014; Urban et al. 2014; Fig. 1). We therefore close with a brief synopsis of those contributions. We hope that the end result is a balanced account of what is currently known about nature of phenotypic responses to climate change.

## Basic considerations

Studies seeking evolutionary inference face the three basic challenges introduced above. One challenge is to understand the proximate causes of phenotypic change: that is, to what degree do the observed shifts reflect genetic and/or plastic changes. Another challenge is to understand the ultimate cause at a basic level: that is, natural selection, sexual selection, genetic drift, or gene flow. The third challenge is to establish the ultimate cause at a specific level: that is, the precise environmental driver, such as commercial fishing, climate change, or pollution.

Each of the above inferences might be desired in synchronic (i.e., spatial) or allochronic (i.e., longitudinal or temporal) contexts. Synchronic studies compare different populations sampled at approximately the same time, whereas allochronic studies compare the same population sampled at different points in time (Hendry and Kinnison 1999). Most inferential methods are easier to implement in the synchronic case because representatives from the different populations can be reared/raised/grown together under specific conditions and because direct assessments of selection can be implemented *in situ*. Studies of contemporary climate change, however, are generally allochronic (i.e., we wish to infer whether a population in the present differs from the same population in the past), a context where inferential methods are harder to implement. In particular, individuals from different times usually cannot be reared/raised/grown together, and past selective regimes are hard to infer with any degree of precision. Similar difficulties attend other allochronic situations, such as evolution in response to commercial fisheries (e.g., Kuparinen and Merilä 2007, 2008) and biological invasions (e.g., Bacigalupe 2009).

If responses to climate change could be assessed synchronically, the present paper would be unnecessary because the various methods have been much discussed and evaluated (e.g., Endler 1986; Kawecki and Ebert 2004; Blanquart et al. 2013). By contrast, methods for inference in allochronic contexts have not been treated in as much detail, including in relation to climate change. In the following sections, we first focus on proximate causes by outlining different approaches for inferring genetic versus plastic changes in allochronic studies. We then address the challenges encountered when attempting to infer ultimate causes in the same context: that is, whether natural selection is the general driver and whether climate change is the specific driver.

An addition point to bear in mind – although not one we here consider in detail – is that phenotypic constancy in the face of environmental change can also represent an important response to climate change – and can be considered in the contexts of ‘phenotypic buffering’ (in the context of plasticity – Reusch 2014) or ‘genetic compensation’ (in the context of genetic change – Conover and Schultz 1995; Grether 2005).

## Genetic or plastic?

### Approaches for inferring genetic change

Six basic approaches might be considered for inferring genetic change: ‘animal model’ analyses, common-garden studies, comparison to model predictions, experimental evolution, space-for-time substitutions, and molecular genetic change (Table 1). Each of these methods has its own strengths and weaknesses, and some allow more robust inferences than do others.

**Table 1:** Synopsis of methods for inferring genetic versus plastic responses to climate change-mediated selection and their adaptive basis. For details, see the main text

**Methods for inferring genetic change**
1. Animal model analyses
2. Common-garden studies
3. Comparisons to model predictions
4. Experimental evolution
5. Space-for-time substitutions
6. Molecular genetic approaches
**Methods for inferring plastic change**
1. Animal model analyses
2. Common-garden studies
3. Experimental studies
4. Fine-grained population responses
5. Individual plasticity in nature
**Methods for inferring the adaptive nature of change**
1. Reciprocal transplant experiments
2. Phenotypic selection estimates
3. Genotypic selection estimates
4. Comparison to neutral expectations
5. Q_ST_–F_ST_ comparisons
**Methods for inferring a specific causal driver**
1. Common sense
2. Environment–trait correlations
3. Experimental selection/evolution

#### Animal model analyses

This quantitative genetic approach is based on temporal changes in estimates of mean breeding values (i.e., ‘genotypic values’) for phenotypic traits. The strength of this approach is that it specifically quantifies genetic shifts while controlling for plasticity (e.g., Réale et al. 2003). A major limitation is that doing so requires pedigree data, which is usually available only in long-term studies of individually marked (or genotyped) vertebrate populations (e.g., Réale et al. 2003; Charmantier et al. 2008). Additional shortcomings can include problems in the estimation and interpretation of changes in mean breeding values. In particular, it sometimes can be difficult to ascertain whether observed trends – or the lack thereof – are biologically meaningful as opposed to methodological artifacts (Postma 2006; Hadfield et al. 2010). When these problems can be overcome by suitable data and appropriate methods, the animal model remains one of the best approaches for evaluating the genetic basis of phenotypic change in a climate change context. In the present special issue, animal models are emphasized in the contributions of Charmantier and Gienapp (2014), Boutin and Lane (2014), and Teplitsky and Millien (2014).

#### Common-garden studies

In order to isolate genetic contributions to trait differences, individuals of known family background can be reared/raised/grown under common laboratory or field conditions (Conover and Schultz 1995; Blanquart et al. 2013). In allochronic contexts, this approach can be implemented in two basic procedures. First, populations for which common-garden studies were conducted in the past can be re-analyzed with new common-garden studies in the present. This approach was used to demonstrate climate-associated genetic shifts in the phenology of pitcher-plant mosquitoes (*Wyeomyia smithii*; Bradshaw and Holzapfel 2001). A difficulty in implementing such studies that the past common-garden environment needs to be very accurately replicated in the new experiment. Second, organisms with dormant stages, such as aquatic invertebrates with resting eggs (e.g., *Daphnia*, Cousyn et al. 2001) or plants with long-lasting seeds (e.g., Franks et al. 2007), can be resurrected from the past for direct comparison to individuals from the present. In either procedure, it is important to control for maternal effects and to recognize that genotype-by-environment interactions can make extrapolation to the natural environment difficult. In the present special issue, allochronic common-garden studies figure most heavily in the contributions of Stoks et al. (2014) and Franks et al. (2014).

#### Comparison to model predictions

This approach compares observed phenotypic trends to predictions from evolutionary models, such as the breeder's equation or its multivariate equivalent (Lynch and Walsh 1998). Specifically, selection and additive genetic (co)variances are measured and used to predict evolutionary responses. If observed trends match predicted trends, then adaptive genetic change is inferred (e.g., Swain et al. 2007; Crozier et al. 2011). Several concerns attend this method. First, it is difficult to accurately measure selection in nature (Kingsolver et al. 2001, 2012; Kruuk et al. 2003; Hersch and Phillips 2004; Morrissey and Hadfield 2012). Second, it is even more difficult to accurately estimate additive genetic (co)variances (Lynch and Walsh 1998), not the least because they vary with environmental conditions (Hoffmann and Merilä 1999). Third, a large number of other assumptions attend these predictive models (Merilä et al. 2001; Morrissey et al. 2010). Given these difficulties, it is perhaps not surprising that predicted and observed evolutionary responses are often (although not always) poorly matched in natural populations (Merilä et al. 2001; Kruuk et al. 2008). In the present special issue, comparisons to model predictions are mentioned by Crozier and Hutchings (2014) and models of evolution in response to climate change are reviewed by Kopp and Matuszewskib (2014).

#### Experimental evolution

The basic idea here is to establish experimental populations exposed to different environmental conditions, such as warming temperature (e.g., Van Doorslaer et al. 2007), increasing CO_2_ (e.g., Collins and Bell 2004), or acidification (e.g., Lohbeck et al. 2012). After a period of evolution under these conditions, the different populations can be compared under common conditions to quantify any genetically based phenotypic change, and they can be genotyped to uncover its genetic underpinnings (Kawecki et al. 2012). A major strength of this approach is that, with appropriate replication and controls, it can confirm evolution in response to a specific environmental factor (discussed in more detail later). One limitation of this approach is that logistical constraints often restrict it to small organisms with short generation times. Another limitation is that it cannot provide confirmation that evolution has actually occurred in natural (as opposed to experimental) populations – it is instead a ‘proof-of-principle’ inferential method. This last concern can be somewhat lessened by conducting experiments with natural populations in natural environments – such as in the case of experimental introductions (Reznick and Ghalambor 2005). In the present special issue, the utility of experimental evolution studies in the context of climate change is especially highlighted by Reusch (2014) and Collins et al. (2004).

#### Space-for-time substitution

Most environmental factors that vary in time also vary in space, and – as explained above – inferences in space (synchronic) are far easier than inferences in time (allochronic). A tempting approach therefore has been to find spatial associations between environmental factors and phenotypes that match the temporal associations for which inferences are desired. If common-garden or reciprocal transplant studies show a genetic basis for phenotypic differences in space, then it might seem reasonable to infer the same for the variation in time. The limitation, of course, is that demonstrating an effect in one situation does not necessarily mean that the same effect holds in another situation (as is the case for experimental evolution), and temporal – as opposed to spatial – environmental changes might be occurring faster than evolution can respond. Fukami and Wardle (2010) provide a good account of the assumptions and potential problems related to space-for-time substitution in ecological studies, many of which are relevant also for evolutionary studies. At present, space-for-time substitutions are the most common – and sometimes only – basis for inference in some taxonomic groups, such as fish (Crozier and Hutchings 2014), marine animals and plants (Reusch 2014), and amphibians and reptiles (Urban et al. 2014), as well as in the context of Bergmann's Rule (Teplitsky and Millien 2014).

#### Molecular genetic approaches

A demonstration of shifts in allele frequency confirms that evolution has occurred – but genomes contain thousands to millions of loci, making shifts at some of them inevitable in all situations. A major difficulty then – much more so than when considering phenotypes – is to determine the specific shifts that are relevant. To this end, investigators usually focus on loci known or thought to influence phenotypes or be under selection, or on closely linked neutral loci (Hoffmann and Daborn 2007; Reusch and Wood 2008). However, because the genetic basis of most traits is not known, it is difficult to know in advance which genes or genomic regions to consider. An alternative is to employ genome scans based on candidate genes or anonymous loci, but their relevance to adaptive phenotypic change often remains uncertain (e.g., Storz 2005). To date, the frequencies of some well-characterized genetic polymorphisms have been shown to shift with climate change (e.g., Umina et al. 2005; Balanyá et al. 2006); but even in these cases, the relevant phenotypic traits and selective mechanisms are not well understood (see more about inferring selection below). In addition to searching for shifts in allele frequencies, comparative analyses of gene expression can be used to search for evidence of genetic effects related to population differentiation (e.g., Roberge et al. 2007), including climate change responses. Advances in genomic technologies, together with new insights into the genetic basis of traits (Olson-Manning et al. 2012; Wolkovich et al. 2012), hold promise for future efforts to infer molecular genetic changes driven by climate change (Franks and Hoffmann 2012). In the present special issue, genetic shifts are considered most often by Schilthuizen and Kellermann (2014) and Franks et al. (2014).

### Approaches for inferring plastic change

We now focus on methods for inferring when plastic changes have contributed to temporal changes in mean phenotype. In doing so, it is important to remember that plasticity can have a genetic basis and can evolve (West-Eberhard 2003) and that both plastic and genetic effects can contribute to a phenotypic trend. The two alternatives are therefore not mutually exclusive but rather potential (co)contributors to a given change: ‘cogradient variation’ provides an illuminating example (Conover and Schultz 1995). Five basic approaches might be considered for inferring plasticity: ‘animal model’ analyses, common-garden studies, experimental manipulations, fine-grained population responses, and measures of individual plasticity (Table 1). As was the case for genetic inferences (above), each approach for inferring plasticity has strengths and weaknesses, and some allow more robust inferences than do others.

#### Animal model analyses

As described above, this quantitative genetic approach can determine the extent to which a phenotypic trend has a quantitative genetic basis. By extension, the portion of the phenotypic trend not explained by genetic change is likely to result from plasticity. Plasticity is thus inferred by excluding or removing genetic effects. In an early example of this approach from a climate change context, Réale et al. (2003) estimated that 62% of the change in parturition date in a population of red squirrels (*Tamiasciurus hudsonicus*) was due to plasticity. Even more dramatically, Charmantier et al. (2008) showed that phenological changes in Great tits (*Parus major*) in Oxford were the result of plasticity, rather than genetic change. Hence, with the caveats and limitations listed earlier, animal model approaches can evaluate plastic versus genetic contributions to phenotypic change. In the present special issue, this approach is considered in the most depth by Charmantier and Gienapp (2014) and Boutin and Lane (2014).

#### Common-garden studies

When a common-garden experiment, as described above, fails to confirm a genetic basis for trait differences, a common conclusion is that the differences observed in nature must result from plasticity. As is the case for animal models, inferences in this approach thus involve excluding or removing genetic effects. Limitations (see also above) include the infeasibility of allochronic common-garden studies for most organisms and the possibility of genotype-by-environment interactions that can make results in a specific common garden misleading with respect to nature (e.g., Winkler and Van Buskirk 2012). These concerns are lessened, although not eliminated, by the use of multiple testing environments, particularly those relevant to the natural situation. In the present special issue, common-garden approaches are particularly common among the studies reviewed by Franks et al. (2014) and Stoks et al. (2014).

#### Experimental manipulations

Whether in the laboratory or the field, the experimental manipulation of an environmental factor, such as temperature, can be used to quantify plasticity. The tempting inference then is that variation in the same factor in nature will produce the same phenotypic response – and so plasticity can explain similar environment–trait associations in nature. This indirect ‘proof-of-principle’ approach is limited in having to assume that the relevant environmental factors have been identified and applied at the correct life history stage. If not, failure to find sufficiently explanatory plastic responses could simply mean that the experimental conditions failed to accurately mimic the natural conditions. Alternatively, strong plastic responses in the laboratory might be swamped in nature by other effects – including ‘countergradient’ genetic differences (Conover and Schultz 1995). For these and probably other reasons, experimental studies often do a poor job of predicting observed trends in natural populations (Wolkovich et al. 2012). In the present special issue, papers paying particular attention to experimental manipulations include Franks et al. (2014), Collins et al. (2004), and Urban et al. (2014).

#### Fine-grained population responses

Environmental factors, such as temperature, do not change in a temporally constant fashion; they instead include a stochastic component that introduces substantial year-to-year variation around any coarse-scale temporal trend. In the present special issue, Crozier and Hutchings (2014) present temperature data that illustrate this phenomenon. This year-to-year environmental variation can be assessed for correlations with year-to-year variation in phenotypes so as to develop a yard-stick for plasticity (e.g., Phillimore et al. 2010) – because genetic changes are not expected to be so fine-grained. An important limitation of this approach is the difficulty in verifying whether the change that took place really was the result of plasticity. Also, the relative importance of factors influencing fine- and coarse-grained variation could be quite different. In the present special issue, taxa where fine-grained population responses are frequently considered include birds (Charmantier and Gienapp 2014), mammals (Boutin and Lane 2014), and fish (Crozier and Hutchings 2014).

#### Individual plasticity in nature

When individual organisms live through multiple occurrences of a given event, investigators can measure within-individual among-event associations between environmental variables and phenotypes. For example, individual birds might breed earlier in warmer years. The resulting estimates of individual plasticity are considerably more natural than those derived from the experimental laboratory-based approach mentioned above. If extrapolation of individual plasticity to the population level can explain temporal trends in mean phenotypes, then plasticity might be considered a sufficient explanation for the population trend (Przybylo et al. 2000; Schiegg et al. 2002; Brommer et al. 2005; Nussey et al. 2005; Reed et al. 2006). A practical limitation of this approach is that it can be applied only in situations where phenotypes of individuals can be tracked across multiple events. Other limitations are that within-individual plasticity might (or might not) be constrained in relation to developmental plasticity, the causes of individual-level and population-level trends might be different, and confirmation of individual plasticity does not rule out the possibility that evolution has also occurred. Several papers in the present special issue discuss the virtues of measuring individual plasticity in nature (e.g., Boutin and Lane 2014; Charmantier and Gienapp 2014).

### Comparison of approaches

Each of the above approaches has been used to infer genetic or plastic responses to climate change, but each also has a series of limitations. An important question thus becomes: ‘Which approaches allow the strongest inferences regarding genetic or plastic effects?’ These inferences can come in two basic forms: evidence for a particular effect (genetic change or plasticity) or evidence against a particular effect. The distinction between these forms of inference is important because evidence for or against a particular effect does not necessarily provide evidence for or against the other effect. In particular, evidence that genetic change has taken place does not by itself indicate that plasticity has been unimportant, just as evidence for plasticity does not by itself indicate that genetic change has been unimportant.

When it comes to genetically based phenotypic change, strong inferences are possible through the use of animal models – although interpretational problems can occur (Postma 2006; Hadfield et al. 2010; Charmantier and Gienapp 2014). However, given that such analyses are possible for only a tiny fraction of the situations where inferences are desired, other approaches are necessary. Common-garden studies also allow strong inferences, although they are possible in only some organisms in allochronic contexts. Specifically, the demonstration of phenotypic differences in a common-garden environment confirms that genetic change has taken place, although genotype-by-environment interactions can complicate extrapolations to nature. Such interactions also mean that the *failure* to demonstrate differences in a common-garden environment is not strictly speaking bullet-proof evidence *against* genetic change. Molecular approaches can provide direct evidence that genetic change has occurred but, again, failure to find such differences does not mean that they are absent (they could be present at some nonsurveyed genomic regions) and links to adaptive phenotypes are often uncertain. The other approaches (comparison to model predictions, experimental evolution, space-for-time replacement) provide supporting evidence that amounts to ‘proof-of-principle’ but they cannot confirm that observed changes in nature have been genetic.

When it comes to plasticity, the problem is different – one cannot directly demonstrate that past changes were plastic. Stated another way, demonstrating that plasticity *could* achieve an observed phenotypic trend does not mean that plasticity actually *did* cause that trend. So the best approach here is to show that plasticity could explain the observed trend while also showing that genetic change cannot. Animal models provide a solid route to this inference – because they can disprove genetic change in a phenotype, while also documenting sufficiently explanatory individual plasticity (Réale et al. 2003; Charmantier et al. 2008). Again, however, this approach will be accessible to only a small number of researchers working on a limited set of organisms. Reasonably strong inferences can also come from common-garden experiments showing that genetic change cannot explain a given phenotypic trend (with the above caveats), in combination with other approaches (experimental studies, fine-grained population responses, or individual plasticity) showing that plasticity could explain them. By themselves, however, these latter approaches fall into the ‘proof-of-principle’ category.

As the foregoing assessment makes clear, no single approach will be a panacea. The best route to strong and robust inference is therefore to use a combination of methods.

## Is the change adaptive?

Establishing that an observed phenotypic shift has a genetic basis is a necessary condition for inferring adaptive evolution in response to climate change, but it is not entirely sufficient because genetic changes might not be adaptive. In particular, genetic drift (Lande 1976), gene flow (Garant et al. 2007), and inbreeding (Keller and Waller 2002) can all cause maladaptive genetic changes. Similarly, establishing that an observed phenotypic shift has a plastic basis does not confirm that the change is adaptive – it might instead be the result of environmental stress. Five basic approaches might be considered for inferring the adaptive nature of phenotypic changes: reciprocal transplants, phenotypic selection estimates, genotypic selection estimates, comparison to neutral expectations, and Q_ST_–F_ST_ comparisons.

### Reciprocal transplants

The most direct test for the adaptive significance of a phenotypic change is to reciprocally transplant individuals between environments (Endler 1986; Kawecki and Ebert 2004; Hereford 2009; Blanquart et al. 2013). This approach is obviously difficult in an allochronic context, but it can be implemented in some situations. In particular, adaptive evolution is confirmed if contemporary genotypes have higher fitness in the contemporary environment than do resurrected genotypes from past environments (e.g., Decaestecker et al. 2007). Given that resurrection is feasible for only a small subset of organisms, an alternative approach is to use laboratory or mesocosm experiments to test contemporary genotypes in simulated present and past environments. Higher fitness in the former implies that adaptation to present environments has reduced fitness (owing to trade-offs) in past environments. On the other hand, failure to find higher fitness in present than in past environments does not mean that adaptive evolution is absent – because fitness trade-offs are not always evident between environments (Bennett and Lenski 2007; Blanquart et al. 2013). Note that this approach can be used (with the usual caveats) to assess the adaptive significance of genetic differences (by using individuals from common-garden experiments), plastic differences (by using individuals from experimental manipulations), and their combination (by using individuals captured from the wild).

### Phenotypic selection estimates

If a given phenotypic change is adaptive, it was likely driven by selection – and so one inferential approach is to measure selection in nature. Specifically, adaptation can be inferred when selection acting during a phenotypic change would be predicted to favor that change (e.g., Swain et al. 2007; Crozier et al. 2011). Note, however, that measuring contemporary selection is not sufficient for inferring past selection – because selection can vary through time (Siepielski et al. 2009; Siepielski et al. 2011). Implementing this approach therefore requires long-term datasets of selection. An additional limitation is that the direction of phenotypic change could correspond to the estimated direction of selection without the two being causally linked (Merilä et al. 2001) – although the estimation of selection on breeding values can reduce this concern (Hadfield et al. 2012; see above). Also, as noted earlier, accurate estimates of selection are extremely difficult to obtain in most natural populations.

### Genotypic selection estimates

Analogous to the phenotypic selection approach, investigators can ask whether observed shifts in allele frequency correspond to selection on those alleles. This selection can be estimated either directly or indirectly. Directly, one can genotype individuals and then measure their fitness (e.g., Fournier-Level et al. 2011) or, if individuals cannot be tracked in this manner, allele frequency differences can be measured between age groups. These approaches are attended by many of the same issues that were discussed immediately above for phenotypic selection. Indirectly, one can test for genomic signatures of past selection (e.g., Storz 2005; Foll and Gaggiotti 2008). Limitations here are that it can be difficult to ascertain the relative contributions of selection as opposed to other factors (e.g., population structure and recombination), and when the past selection took place (Barrett and Hoekstra 2011; Nachman and Payseur 2012). An additional limitation of these genotypic approaches is that selection at the genotypic level does not necessarily reflect selection at the phenotypic level. For example, selection on polygenic traits can be very difficult to detect at the individual loci that influence those traits (McKay and Latta 2002; Le Corre and Kremer 2012).

### Comparison to model predictions

A long tradition in evolutionary biology – particularly in paleontology – has been the use of null models of evolutionary change (e.g., Brownian motion or genetic drift) to infer the role (or not) of natural selection (review: Sheets and Mitchell 2001). Specifically, natural selection is typically inferred when the rate or directionality of change exceeds the confidence bounds of a null model. A limitation of this approach is its low inferential power even when strong external evidence supports the role of selection (Bell et al. 2006). More recently, increased inferential power has come from formally comparing alternative models with and without natural selection (e.g., Clegg et al. 2008; Hunt et al. 2008). This null model approach is not very common in the context of contemporary climate change, although Crozier et al. (2011) might be considered in this class.

### Q_ST_–F_ST_ comparisons

This approach is also rooted in null model thinking. Specifically, theory predicts that selection will cause patterns of quantitative trait variation to differ from patterns of neutral marker variation (Lande 1992; McKay and Latta 2002; Leinonen et al. 2008; Whitlock 2008). In particular, divergent selection is often inferred when the among-population proportion of genetically based trait variance (Q_ST_) exceeds the among-population proportion of neutral marker divergence (F_ST_). This approach has a number of limitations. Of particular concern, the accurate estimation of Q_ST_ requires a common-garden study (e.g., Leinonen et al. 2008), which is difficult to implement in an allochronic context. Also in play is the previously noted problem that genotype-by-environment interactions can make inferences misleading with respect to natural populations. A partial solution to this problem can come from using natural populations to estimate the phenotypic Q_ST_ analog (P_ST_: Leinonen et al. 2006), although interpretation remains difficult (Brommer 2011). Many additional concerns attend Q_ST_–F_ST_ comparisons (Hendry 2002; Leinonen et al. 2008; Edelaar et al. 2011), although some of these can be reduced through careful planning or by adopting improved approaches (e.g., Ovaskainen et al. 2011; Karhunen et al. 2013). To date, however, Q_ST_–F_ST_ comparisons have not been much used in the context of contemporary climate change.

## Comparison of approaches

Reciprocal transplants are the most direct approach for inferring adaptive change and should be employed whenever possible. When this approach cannot be applied, the next resort should be methods described in the following section – because these methods can reveal not only that adaptation has likely occurred (the present question) but also the specific environmental driver.

## What is the specific selective force?

Even if the overall adaptive significance of a phenotypic trend has support from the above approaches, the specific environmental driver remains to be established (Wade and Kalisz 1990; MacColl 2011). This remaining question is of particular concern because climate change is often correlated with other environmental changes that might influence phenotypic change, such as changing rates of exploitation by humans, increasing or decreasing pollution, habitat loss and degradation, and increasing impact from invasive species. Inferring that climate change, or a specific aspect of climate change (e.g., temperature, pH), is the causal force thus requires additional effort. Three basic routes to such inferences are possible: common sense, phenotype–environment correlations, and experimental selection/evolution.

### Common sense

The easiest, and certainly still the most common, approach for inferring a specific environmental driver is through existing knowledge and intuition. For example, if (1) organisms reproduce when particular resources become available in the spring, (2) climate change is causing those resources to become available earlier, and (3) the organisms are reproducing earlier, we might feel safe in assuming that climate change is the cause of the observed phenotypic change. Although intuition often might be correct, it is not inevitably or always simplistically so. For example, climate warming is generally assumed to advance the spring phenology of temperate organisms, whereas it actually retards the emergence of alpine ground squirrels (*Urocitellus columbianus*; Lane et al. 2012). At the end of the day, any adaptive ‘story telling’ (*sensu* Gould and Lewontin 1979) should be accompanied by formal hypothesis testing using the following methods.

### Phenotype–environment correlations

Another common inferential approach is to *quantify* the association between trait change and specific aspects of climate change (e.g., Quinn and Adams 1996; Crozier et al. 2011). If a tight correlation is found, especially across multiple independent locations/populations, support for that environmental driver is enhanced. Ideally, multiple potential drivers would be simultaneously assessed so as to determine their independent and interactive effects. A difficulty in implementing this approach is that evolution, and sometimes plasticity, cannot perfectly track environmental change (Quinn and Adams 1996; Both and Visser 2001), and so the failure to find a tight trait–environment association does not necessarily mean that the environmental factor is unimportant. This problem can be partially circumvented by also adding a complementary space-for-time replacement (e.g., Phillimore et al. 2010). If, for instance, temperature is suspected to be the primary factor driving temporal changes in phenotype, similar associations might be expected across spatial temperature gradients. Of course, we return here to the above-mentioned concern that temporal and spatial drivers might not be the same (see also Fukami and Wardle 2010).

### Experimental selection/evolution

Experimental studies are often deemed necessary to conclusively isolate the role of a particular environmental factor in shaping a particular ecological or evolutionary response (Bender et al. 1984). The best manifestation in the context of climate change would be to experimentally manipulate a specific candidate driver in nature, such as through artificial warming of the environment experience by a natural population. With appropriate replication and controls, and following the confirmation of genetic or plastic change and its adaptive significance (as above), exceptionally strong evidence thus would be provided for causal effects. This approach is obviously difficult to apply in nature, and so most such studies instead use controlled laboratory or mesocosm settings (e.g., Van Doorslaer et al. 2007). Although such studies are extremely informative, their relevance to nature is sometimes uncertain. And, of course, experimental manipulations cannot conclusively confirm the specific driver of phenotypic/genetic changes in other (nonexperimental) populations – although ambiguity is lessened if responses are consistent across replicates/contexts and are very similar to those in natural populations.

## Comparison of approaches

As opposed to the previous questions, it is not particularly helpful to here rank the different approaches for the quality of inference they can provide – because each has major inferential limitations. Experimental studies are obviously the best way to demonstrate causation but these are difficult to implement in natural populations and can only conclusively demonstrate causation within the experiment itself. The other approaches are more relevant to natural populations, but are correlational. Thus, the most convincing cases are built based on combined evidence from observational and experimental studies.

## Where are we now and what comes next?

Our goal in preparing the present paper was to provide a thorough and honest appraisal of the different approaches for inferring genetic and plastic responses to climate change. Having done so, we asked experts to use our appraisal as a basis for evaluating the evidence for such changes in specific taxa and contexts. In the rest of this special issue, these assessments are provided for bird breeding and migration timing (Charmantier and Gienapp 2014), mammals (Boutin and Lane 2014), fish (Crozier and Hutchings 2014), amphibians and reptiles (Urban et al. 2014), terrestrial plants (Franks et al. 2014), marine plants and animals (Reusch 2014), terrestrial arthropods (Schilthuizen and Kellermann 2014), and freshwater arthropods (Stoks et al. 2014). We also asked for a similar assessment for Bergmann's rule in a climate change context (Teplitsky and Millien 2014) and for a state-of-the art summary of theoretical approaches (Kopp and Matuszewskib 2014). Although these targeted papers should be consulted for details, we now provide a brief summary of their findings.

Even a cursory reading of the special issue reinforces the view that our understanding of genetic and plastic responses to climate change is still in its infancy. In particular, although a huge number of studies have found evidence of climate-associated phenotypic trends (e.g., Parmesan and Yohe 2003), only a few have used the best methods for inferring genetic versus plastic change, adaptive versus nonadaptive responses, and specific environmental drivers. Focusing specifically on studies using strong methods of inference for the first of these topics provides a soberingly short list (Table 2). That is, instances of confirmed genetic change do occur in birds and mammals, and especially in terrestrial plants and insects, but are quite few. By contrast, considerably more studies have found evidence for plastic contributions – as summarized by the various contributions to this special issue. These findings are highlighted by the results of Teplitsky and Millien (2014) who focused on temporal body size clines in vertebrates: None of studies listed in their Table 2 provide evidence for genetic basis of observed body size changes.

**Table 2:** A synopsis of studies testing for climate-driven genetic changes in an allochronic (temporal) context in nature. Most of these studies are drawn from the taxonomic reviews in the present special issue – to which citations are provided. Owing to our specific focus (genetic change, allochronic studies, and natural populations), we here exclude space-for-time substitutions and experimental evolution in the laboratory. Also for this reason, no studies of amphibians or reptiles (Urban et al. 2014), marine phytoplankton (Collins et al. 2004), or other marine organisms (Reusch 2014) appear in the table. Also indicated is whether the adaptive nature of the change has been confirmed and whether climate change has been established as a causal factor. The numbers in parentheses refer to approaches listed in Table 1. ‘Yes’ = evidence provided, ‘No’ = no evidence, ‘.’ = not investigated

Species	Trait	Genetic change?	Plastic change?	Adaptive?	Causality?	Reference
**Fish**						
*Oncorhynchus gorbuscha*	Phenology	Y (6)	.	.	Y (1,2)	Crozier and Hutchings 2014
*Oncorhynchus nerka*	Phenology	Y (3)	Y (4)	Y (2)	Y (1,2)	Crozier and Hutchings 2014
**Birds**						
*Larus novaehollandiae*	Body size	N (1)	Y (5)	N (2,3)	N (2)	Teplitsky and Millien 2014
	Phenology	N (1)	N (5)	N (2,3)	.	Charmantier and Gienapp 2014
*Strix aluco*	Coloration	Y (1)	N (5)	Y (2)	Y (2)	Karell et al. 2011
*Ficedula albicollis*	Phenology	N (1)	Y (5)	Y (2,3)	Y (2)	Charmantier and Gienapp 2014
*Parus major*	Phenology	N (1,5)	Y (5)	Y (2,3)	Y (2)	Charmantier and Gienapp 2014
	Body size	N (1)	Y (5)	Y (2,3)	Y (2)	Teplitsky and Millien 2014
*Sylvia atricapilla*	Phenology	Y (2,5)	.	Y (2,3)	Y (1–3)	Charmantier and Gienapp 2014
**Mammals**						
*Tamiasciurus hudsonicus*	Phenology	Y (1,3)	Y (1,4,5)	Y (2)	Y (2)	Boutin and Lane 2014
*Marmota flaviventris*	Phenology Body size	N (1)	Y (1)	Y (2)	Y (1)	Boutin and Lane 2014
*Ovis aries*	Body size	N (1)	Y (1,4,5)	Y (2)	Y (2)	Boutin and Lane 2014
**Plants**						
*Brassica rapa*	Phenology Physiology	Y (2,3)	Y (4), N (2,3)	Y (1,2)	Y (2,3)	Franks et al. 2014
*Thymus vulgaris*	Allele frequency	Y (6)	.	Y (2)	Y (1)	Franks et al. 2014
*Andropogon gerardii*	Physiology, growth	Y (2,3,6)	Y (2,3,4)	Y (3)	Y (3)	Franks et al. 2014
*Triticum dicoccoides* & *Hordeum spontaneum*	Phenology, allele frequency	Y (2,6)	.	.	Y (2)	Franks et al. 2014
*Polygonum cespitosum*	Physiology, growth	Y (2,3)	Y (2,3)	Y (1,2)	Y (2,3)	Franks et al. 2014
**Insects**						
*Aedes albopictus*	Phenology	Y (2,5)	.	.	.	Stoks et al. 2014
*Wyeomyia smithii*	Phenology	Y (2,5)	.	Y (1)	Y (1)	Stoks et al. 2014
*Aquarius paludum*	Phenology	Y (2)	.	.	Y (1)	Stoks et al. 2014
*Hesperia comma*	Dispersal traits	Y (2)	N (2)	.	Y (2)	Schilthuizen and Kellermann 2014
*Aricia agestis*	Dispersal traits	Y (2)	N (2)	.	Y (2)	Schilthuizen and Kellermann 2014
*Adalia bipunctata*	Coloration	Y (2)	N (2)	Y (2,3)	Y (2)	Schilthuizen and Kellermann 2014
*Tetrix undulata*	Dispersal traits	Y (2)	N (2)	N (2)	.	Schilthuizen and Kellermann 2014
*Drosophila melanogaster*	Allele frequency	Y (6)	.	.	Y (1)	Schilthuizen and Kellermann 2014
*Drosophila subobscura*	Allele frequency	Y (6)	.	.	Y (1)	Schilthuizen and Kellermann 2014
*Drosophila robusta*	Allele frequency	Y (6)	.	.	Y (1)	Schilthuizen and Kellermann 2014

Overall, it seems safe to conclude that plasticity often makes a strong contribution to phenotypic trends associated with contemporary climate change. Genetic contributions, however, seem to be weaker and less common – although the reality is that only a few definitive tests have been performed. It may be that application of better inferential methods will uncover many more examples of genetic change (many such examples are certainly known in spatial contexts), or it may be that not enough time has passed for substantial evolution to take place. Although this constraint might well be important, plenty of examples certainly do exist of genetically based adaptation to local temperature differences on similar time frames, including in taxa for which few examples appear in Table 2. For instance, the contemporary evolution of temperature-dependent development has been shown for salmonid fish populations introduced to new thermal environments (e.g., Haugen and Vøllestad 2000) and for amphibian populations subject to pond warming as a result of beaver activity (Skelly and Freidenburg 2000). Evolutionary responses to climate change therefore demands considerably more study.

To sum up, this perspective and the accompanying eleven articles have focused on methods and quality of evidence for genetic and phenotypic changes to climate change in nature. While the current picture emerging from all of this work might not seem particularly encouraging, it should provide guidelines, avenues, and inspiration for research to come. Identification of the challenges and knowledge gaps can be viewed as first step toward progress in improving our understanding of the relative roles of genetic change and plasticity in mediating adaptive organismal responses to changing climatic conditions.

## References

[b1] Anderson JT, Wagner MR, Rushworth CA, Prasad KVSK, Mitchell-Olds T (2013). The evolution of quantitative traits in complex environments. Heredity.

[b2] Bacigalupe LD (2009). Biological invasions and phenotypic evolution: a quantitative genetic perspective. Biological Invasions.

[b3] Balanyá J, Oller JM, Huey RB, Gilchrist GW, Serra L (2006). Global genetic change tracks global climate warming in *Drosophila subobscura*. Science.

[b4] Barrett RD, Hoekstra HE (2011). Molecular spandrels: test of adaptation at the genetic level. Nature Reviews Genetics.

[b5] Bell MA, Travis MP, Blouw DM (2006). Inferring natural selection in a fossil threespine stickleback. Paleobiology.

[b6] Bender EA, Case TJ, Gilpin ME (1984). Perturbation experiments in community ecology – theory and practice. Ecology.

[b7] Bennett AF, Lenski RE (2007). An experimental test of evolutionary trade-offs during temperature adaptation. Proceedings of the National Academy of Sciences U.S.A.

[b8] Blanquart F, Kaltz O, Nuismer S, Gandon S (2013). A practical guide to measuring local adaptation. Ecology Letters.

[b9] Both C, Visser ME (2001). Adjustment to climate change is constrained by arrival date in a long-distance migrant bird. Nature.

[b10] Boutin S, Lane JE (2014). Climate change and mammals: evolutionary versus plastic responses. Evolutionary Applications.

[b11] Bradshaw WE, Holzapfel CM (2001). Genetic shift in photoperiodic response correlated with global warming. Proceedings of the National Academy of Sciences U.S.A.

[b12] Brommer JE (2011). Whither P_ST_? The approximation of Q_ST_ by P_ST_ in evolutionary and conservation biology. Journal of Evolutionary Biology.

[b13] Brommer JE, Merilä J, Sheldon BC, Gustafsson L (2005). Natural selection and genetic variation for reproductive reaction norms in a wild bird population. Evolution.

[b14] Bürger R, Lynch M (1995). Evolution and extinction in a changing environment: a quantitative-genetic analysis. Evolution.

[b15] Charmantier A, Gienapp P (2014). Climate change and timing of avian breeding and migration: evolutionary versus plastic responses. Evolutionary Applications.

[b16] Charmantier A, McCleery RH, Cole LR, Perrins C, Kruuk LEB, Sheldon B (2008). Adaptive phenotypic plasticity in response to climate change in a wild bird population. Science.

[b17] Clegg SM, Frentiu FD, Kikkawa J, Tavecchia G, Owens IPF (2008). 4000 Years of phenotypic change in an island bird: heterogeneity of selection over three microevolutionary timescales. Evolution.

[b18] Collins S, Bell G (2004). Phenotypic consequences of 1000 generations of selection at elevated CO2 in a green alga. Nature.

[b119] Collins S, B Rost, Rynearson TA (2014). Evolutionary potential of marine phytoplankton under ocean acidification. Evolutioanry Applications.

[b19] Conover D, Schultz ET (1995). Phenotypic similarity and the evolutionary significance of countergradient variation. Trends in Ecology and Evolution.

[b20] Cousyn C, De Meester L, Colbourne JK, Brendonck L, Verschuren D, Volckaert F (2001). Rapid, local adaptation of zooplankton behavior to changes in predation pressure in the absence of neutral genetic changes. Proceedings of the National Academy of Sciences of the United States of America.

[b21] Crozier LG, Hutchings JA (2014). Plastic and evolutionary responses to climate change in fish. Evolutionary Applications.

[b22] Crozier LG, Scheuerell MD, Zabel RW (2011). Using time series analysis to characterize evolutionary and plastic responses to environmental change: a case study of a shift toward earlier migration date in sockeye salmon. American Naturalist.

[b23] Decaestecker E, Gaba S, Raeymaekers JAM, Stoks R, Van Kerckhoven L, Ebert D, De Meester L (2007). Host–parasite ‘Red Queen’ dynamics archived in pond sediment. Nature.

[b24] Edelaar P, Burraco P, Gomez-Mestre I (2011). Comparisons between Q_ST_ and F_ST_ –how wrong have we been?. Molecular Ecology.

[b25] Ellegren H, Sheldon BC (2008). Genetic basis of fitness differences in natural populations. Nature.

[b26] Endler JA (1986). Natural Selection in the Wild.

[b27] Foll M, Gaggiotti O (2008). A genome-scan method to identify selected loci appropriate for both dominant and codominant markers: a Bayesian perspective. Genetics.

[b28] Fournier-Level A, Korte A, Cooper MD, Nordborg M, Schmitt J, Wilczek AM (2011). A map of local adaptation in *Arabidopsis thaliana*. Science.

[b29] Franks SJ, Hoffmann AA (2012). Genetics of climate change adaptation. Annual Review of Genetics.

[b30] Franks SJ, Sim S, Weis AE (2007). Rapid evolution of flowering time by an annual plant in response to a climate fluctuation. Proceedings of the National Academy of Sciences of the United States of America.

[b31] Franks SJ, Weber JJ, Aitken SN (2014). Evolutionary and plastic responses to climate change in terrestrial plant populations. Evolutionary Applications.

[b32] Fukami T, Wardle DA (2010). Long-term ecological dynamics: reciprocal insights from natural and anthropogenic gradients. Proceedings of the Royal Society B: Biological Sciences.

[b33] Garant D, Forde SE, Hendry AP (2007). The multifarious effects of dispersal and gene flow on contemporary adaptation. Functional Ecology.

[b34] Gienapp P, Teplitsky C, Alho JS, Mills JA, Merilä J (2008). Climate change and evolution: disentangling environmental and genetic responses. Molecular Ecology.

[b35] Gotthard K, Nylin S (1995). Adaptive plasticity and plasticity as an adaptation: a selective review of plasticity in animal morphology and life-history. Oikos.

[b36] Gould SJ, Lewontin RC (1979). The spandrels of San Marco and the Panglossian paradigm: a critique of the adaptationist programme. Proceedings of the Royal Society B: Biological Sciences.

[b37] Grether GF (2005). Environmental change, phenotypic plasticity, and genetic compensation. American Naturalist.

[b38] Hadfield JD, Wilson AJ, Garant D, Sheldon BC, Kruuk LE (2010). The misuse of BLUP in ecology and evolution. American Naturalist.

[b39] Haugen TO, Vøllestad AL (2000). Population differences in early life-history traits in grayling. Journal of Evolutionary Biology.

[b40] Hendry AP (2002). QST > = ≠ < FST?. Trends in Ecology and Evolution.

[b41] Hendry AP, Kinnison MT (1999). The pace of modern life: measuring rates of contemporary microevolution. Evolution.

[b42] Hendry AP, T. J Farrugia, Kinnison MT (2008). Human influences on rates of phenotypic change in wild animal populations. Molecular Ecology.

[b43] Hereford J (2009). A quantitative survey of local adaptation and fitness trade-offs. American Naturalist.

[b44] Hersch EI, Phillips PC (2004). Power and potential bias in field studies of natural selection. Evolution.

[b45] Hoffmann AA, Daborn PJ (2007). Towards genetic markers in animal populations as biomonitors for human-induced environmental change. Ecology Letters.

[b46] Hoffmann AA, Merilä J (1999). Heritable variation and evolution under favourable and unfavourable conditions. Trends in Ecology and Evolution.

[b47] Holt RD (1990). The microevolutionary consequences of climate change. Trends in Ecology and Evolution.

[b48] Hunt G, Bell MA, Travis MP (2008). Evolution toward a new adaptive optimum: phenotypic evolution in a fossil stickleback lineage. Evolution.

[b49] James FC (1983). Environmental component of morphological differentiation in birds. Science.

[b50] Karell P, Ahola K, Karstinen T, Valkama J, Brommer JE (2011). Climate change drives microevolution in a wild bird. Nature Communications.

[b51] Karhunen M, Merilä J, Leinonen T, Cano JM, Ovaskainen O (2013). driftsel: an R package for detecting signals of natural selection in quantitative traits. Molecular Ecology Resources.

[b52] Kawecki TJ, Ebert D (2004). Conceptual issues in local adaptation. Ecology Letters.

[b53] Kawecki TJ, Lenski RE, Ebert D, Hollis B, Olivieri I, Whitlock MC (2012). Experimental evolution. Trends in Ecology and Evolution.

[b54] Keller LF, Waller DM (2002). Inbreeding effects in wild populations. Trends in Ecology and Evolution.

[b55] Kingsolver JG, Hoekstra HE, Hoekstra JM, Berrigan D, Vignieri SN, Hill CE, Hoang A (2001). The strength of phenotypic selection in natural populations. American Naturalist.

[b56] Kingsolver JG, Diamond SE, Siepielski AM, Carlson SM (2012). Synthetic analyses of phenotypic selection in natural populations: lessons, limitations and future directions. Evolutionary Ecology.

[b57] Kopp M, Matuszewskib S (2014). Rapid evolution of quantitative traits: theoretical perspectives. Evolutionary Applications.

[b58] Kruuk LEB, Merilä J, Sheldon BC (2003). When environmental variation short-circuits natural selection. Trends in Ecology and Evolution.

[b59] Kruuk LEB, Slate J, Wilson AJ (2008). New answers for old questions: the evolutionary quantitative genetics of wild animal populations. Annual Review of Ecology, Evolution, and Systematics.

[b60] Kuparinen A, Merilä J (2007). Detecting and managing fisheries-induced evolution. Trends in Ecology and Evolution.

[b61] Kuparinen A, Merilä J (2008). The role of fisheries-induced evolution. Science.

[b62] Lande R (1976). Natural selection and random genetic drift in phenotypic evolution. Evolution.

[b63] Lande R (1992). Neutral theory of quantitative genetic variance in an island model with local extinction and colonization. Evolution.

[b64] Lane JE, Kruuk LEB, Charmantier A, Murie JO, Dobson FS (2012). Delayed phenology and reduced fitness associated with climate change in a wild hibernator. Nature.

[b65] Le Corre V, Kremer A (2012). The genetic differentiation at quantitative trait loci under local adaptation. Molecular Ecology.

[b66] Leinonen T, Cano JM, Mäkinen H, Merilä J (2006). Contrasting patterns of body shape and neutral genetic divergence in marine and lake populations of threespine sticklebacks. Journal of Evolutionary Biology.

[b67] Leinonen T, O'Hara RB, Cano JM, Merilä J (2008). Comparative studies of quantitative trait and neutral marker divergence: a meta-analysis. Journal of Evolutionary Biology.

[b68] Lohbeck KT, Riebesell U, Reusch TBH (2012). Adaptive evolution of a key phytoplankton species to ocean acidification. Nature Geoscience.

[b69] Lynch M, Lande R, Kareiva PM, Kingsolver JG, Huey RB (1993). Evolution and extinction in response to environmental change. Biotic Interactions and Global Change.

[b70] Lynch M, Walsh B (1998). Genetics and Analysis of Quantitative Traits.

[b71] MacColl ADC (2011). The ecological causes of evolution. Trends in Ecology and Evolution.

[b72] Mackay TFC, Stone EA, Ayroles JF (2009). The genetics of quantitative traits: challenges and prospects. Nature Reviews Genetics.

[b73] McKay JK, Latta RG (2002). Adaptive population divergence: markers, QTL and traits. Trends in Ecology and Evolution.

[b74] Merilä J (2012). Evolution in response to climate change: in pursuit of the missing evidence. BioEssays.

[b75] Merilä J, Sheldon BC, Kruuk LEB (2001). Explaining stasis: microevolutionary studies in natural populations. Genetica.

[b76] Morrissey MB, Hadfield JD (2012). Directional selection in temporally replicated studies is remarkably consistent. Evolution.

[b77] Morrissey MB, Kruuk LEB, Wilson AJ (2010). The danger of applying the breeder's equation in observation studies of natural populations. Journal of Evolutionary Biology.

[b78] Nachman MW, Payseur BA (2012). Recombination rate variation and speciation: theoretical predictions and empirical results in rabbits and mice. Proceedings of the Royal Society B Biological Sciences.

[b79] Nussey DH, Postma E, Gienapp P, Visser ME (2005). Selection on heritable phenotypic plasticity in a wild bird population. Science.

[b80] Olson-Manning CF, Wagner MR, Mitchell-Olds T (2012). Adaptive evolution: evaluating empirical support for theoretical predictions. Nature Reviews in Genetics.

[b81] Ovaskainen O, Karhunen M, Zheng CZ, Arias JMC, Merilä J (2011). A new method to uncover signatures of divergent and stabilizing selection in quantitative traits. Genetics.

[b82] Parmesan C, Yohe G (2003). A globally coherent fingerprint of climate change impacts across natural systems. Nature.

[b83] Phillimore AB, Hadfield JD, Jones OR, Smithers RJ (2010). Differences in spawning date between populations of common frog reveal local adaptation. Proceedings of the National Academy of Sciences of the United States of America.

[b84] Postma E (2006). Implications of the difference between true and predicted breeding values for the study of natural selection and micro-evolution. Journal of Evolutionary Biology.

[b85] Przybylo R, Sheldon BC, Merilä J (2000). Climatic effects on breeding and morphology: evidence for phenotypic plasticity. Journal of Animal Ecology.

[b86] Quinn TP, Adams DC (1996). Environmental changes affecting the migratory timing of American shad and sockeye salmon. Ecology.

[b87] Réale D, McAdam AG, Boutin S, Berteaux D (2003). Genetic and plastic responses of a northern mammal to climate change. Proceedings of the Royal Society B: Biological Sciences.

[b88] Reed TE, Wanless S, Harris MP, Fredriksen M, Kruuk LEB, Cunningham EJA (2006). Responding to environmental change: plastic responses vary little in a synchronous breeder. Proceedings of Royal Society B: Biological Sciences.

[b89] Reusch TBH (2014). Climate change in the oceans: evolutionary versus. phenotypically plastic responses. Evolutionary Applications.

[b90] Reusch TBH, Wood TE (2008). Molecular ecology of global change. Molecular Ecology.

[b91] Reznick DN, Ghalambor CK (2005). Selection in nature: experimental manipulations in natural populations. Integrative and Comparative Biology.

[b92] Roberge C, Guderley H, Bernatchez L (2007). Genome-wide identification of genes under selection: gene transcription Qst scan in diverging Atlantic salmon subpopulations. Genetics.

[b94] Schiegg K, Pasinelli G, Walters JR, Daniels SJ (2002). Inbreeding and experience affect response to climate change by endangered woodpeckers. Proceedings of the Royal Society of London B: Biological Sciences.

[b95] Schilthuizen M, Kellermann V (2014). Contemporary climate change and terrestrial invertebrates: evolutionary versus plastic changes. Evolutionary Applications.

[b96] Sheets HD, Mitchell CM (2001). Why the null matters: statistical tests, random walks and evolution. Genetica.

[b97] Siepielski AM, DiBattista JD, Carlson SM (2009). It's about time: the temporal dynamics of phenotypic selection in the wild. Ecology Letters.

[b98] Siepielski AM, DiBattista JD, Evans JA, Carlson SM (2011). Differences in the temporal dynamics of phenotypic selection among fitness components in the wild. Proceedings B. Biological Sciences.

[b99] Skelly DK, Freidenburg LK (2000). Effects of beaver on the thermal biology of an amphibian. Ecology Letters.

[b100] Stoks R, Geerts AN, De Meester L (2014). Evolutionary and plastic responses of freshwater invertebrates to climate change: realized patterns and future potential. Evolutionary Applications.

[b101] Storz JF (2005). Using genome scans of DNA polymorphism to infer adaptive population divergence. Molecular Ecology.

[b102] Swain DP, Sinclair AF, Mark Hanson J (2007). Evolutionary response to size-selective mortality in an exploited fish population. Proceedings of the Royal Society B: Biological Sciences.

[b103] Teplitsky C, Millien V (2014). Climate warming and Bergmann's rule through time: is there any evidence?. Evolutionary Applications.

[b104] Teplitsky C, Mills JA, Alho JS, Yarall JW, Merilä J (2008). Bergmann's Rule and climate change: disentangling environmental and genetic responses in a wild bird population. Proceedings of the National Academy of Sciences U. S. A.

[b106] Umina PA, Weeks AR, Kearney MR, McKechnie SW, Hoffmann AA (2005). A rapid shift in a classic clinal pattern in *Drosophila* reflecting climate change. Science.

[b107] Urban M, Richardson J, Freidenfelds N (2014). Plasticity and genetic adaptation mediate amphibian and reptile responses to climate change. Evolutionary Applications.

[b108] Van Doorslaer W, Stoks R, Jeppesen E, De Meester L (2007). Adaptive microevolutionary responses to simulated global warming in *Simocephalus vetulus*: a mesocosm study. Global Change Biology.

[b109] Wade MJ, Kalisz S (1990). The causes of natural selection. Evolution.

[b110] West-Eberhard MJ (2003). Developmental Plasticity and Evolution.

[b111] Whitlock MC (2008). Evolutionary inference from Q_ST_. Molecular Ecology.

[b112] Winkler JD, Van Buskirk J (2012). Influence of experimental venue on phenotype: multiple traits reveal multiple answers. Functional Ecology.

[b113] Wolkovich EM, Cook BI, Allen JM, Crimmins TM, Betancourt JL, Travers SE, Pau S (2012). Warming experiments underpredict plant phenological responses to climate change. Nature.

